# *Candida auris* Discovery through Community Wastewater Surveillance during Healthcare Outbreak, Nevada, USA, 2022

**DOI:** 10.3201/eid2902.221523

**Published:** 2023-02

**Authors:** Alessandro Rossi, Jorge Chavez, Thomas Iverson, John Hergert, Kelly Oakeson, Nathan LaCross, Chidinma Njoku, Andrew Gorzalski, Daniel Gerrity

**Affiliations:** Author affiliations: Utah Department of Health and Human Services, Salt Lake City, Utah, USA (A. Rossi, J. Chavez, T. Iverson, J. Hergert, K. Oakeson, N. LaCross); Nevada Department of Health and Human Services, Las Vegas, Nevada, USA (C. Njoku); University of Nevada, Reno, Nevada, USA (A. Gorzalski); Southern Nevada Water Authority, Las Vegas (D. Gerrity).

**Keywords:** *Candida auris*, candidiasis, antimicrobial resistance, wastewater, nosocomial infections, outbreaks, fungi, yeasts, Nevada, United States

## Abstract

*Candida auris* transmission is steadily increasing across the United States. We report culture-based detection of *C. auris* in wastewater and the epidemiologic link between isolated strains and southern Nevada, USA, hospitals within the sampled sewershed. Our results illustrate the potential of wastewater surveillance for containing *C. auris*.

The COVID-19 pandemic has showcased wastewater surveillance as an effective and economical tool for monitoring disease transmission levels and guiding public health interventions (*1**–**2*). Wastewater surveillance could also conceivably contribute to early detection of drug-resistant organisms of high relevance, such as carbapenem-resistant Enterobacterales and the yeast *Candida auris* (*3*). Carbapenem-resistant Enterobacterales have been recovered from influent of wastewater treatment plants (WWTPs), as well as from effluent from healthcare facilities (*4**–**5*). Various fungi and yeast species have also been cultured from wastewater (*6*). 

*C. auris* was first described at the species level in 2009 and quickly became a notable nosocomial pathogen, displaying high levels of resistance to azoles and, to a lesser extent, polyenes and echinocandins (*7*). Limited treatment options, substantial pathogenicity, and environmental persistence in healthcare settings define *C. auris* as one of the most formidable public health threats in the world (*7*). Epidemiologically, *C. auris* isolates are assigned to 5 distinct genomic clades describing their original endemicity patterns: I (southern Asia), II (eastern Asia), III (Africa), IV (South America), and V (Iran) (*7**,**8*). We illustrate the potential utility of community-level wastewater surveillance for *C. auris* through culture-based monitoring at 2 WWTPs in southern Nevada, USA, while they were receiving sewage from healthcare facilities experiencing an outbreak. 

## The Study 

In Nevada, the first clinical case of *C. auris* was identified in August 2021. As of June 2022, a total of 300 cases had been reported and 22 healthcare facilities affected, including 10 acute care hospitals, 8 skilled nursing facilities, and 4 long-term acute care facilities. Clade III *C. auris* comprises most cases in Nevada, but clades I, II, and IV have also been identified. This sustained pathogen transmission represented an ideal scenario to explore the potential of wastewater surveillance for *C. auris*. 

We collected wastewater influent samples (i.e., raw sewage) from 2 WWTPs (WWTP1 and WWTP2) in southern Nevada. WWTP1 treats an average daily flow of ≈100 million gallons/day; it serves a local population of nearly 1 million people, in addition to ≈800,000 weekly visitors. WWTP2 treats an average daily flow of ≈5 million gallons/day and serves a primarily residential sewershed with ≈90,000 people. We collected 50 mL aliquots of wastewater influent grab samples from each facility on the mornings of May 23 and 31 and June 6 and 13, 2022. We shipped samples on ice overnight to the Utah Public Health Laboratory, Utah Department of Health and Human Services (Taylorsville, UT, USA), where they were processed immediately upon receipt. We centrifuged each 50 mL sample at 5,000 × *g* for 10 minutes and resuspended the resulting pellet in 1 mL of 0.9% saline. We used 100 μL of this suspension to inoculate 2 mL of S^2^ Media salt Sabouraud dulcitol broth (SSDB; Thomas Scientific, https://www.thomassci.com) (*9*) containing fluconazole (SSDBF) at a final concentration of 8 μg/mL. We incubated inoculated SSDBF tubes at 42°C for up to 7 d with vigorous agitation at 250 rpm. After incubation, we plated 100 μL of the inoculated SSDBF broth on HardyCHROM Candida medium (Hardy Diagnostics, https://hardydiagnostics.com) or BBL CHROMagar Candida medium (Becton, Dickinson and Company, https://www.bd.com). Alternatively, we used a 1 μL loop for quadrant streaking. We used matrix-assisted laser desorption/ionization time-of-flight mass spectrometry (Bruker, https://www.bruker.com) to identify species of growing colonies. We performed whole-genome sequencing on NextSeq 2000 or NovaSeq platforms (Illumina, https://www.illumina.com), and performed genome assembly as described elsewhere (*10*). We mapped reads to a reference *C. auris* genome and performed single-nucleotide polymorphism (SNP) and phylogenetic tree analyses using the MycoSNP pipeline (https://github.com/CDCgov/mycosnp-nf) (*10*). 

**Figure 1:**
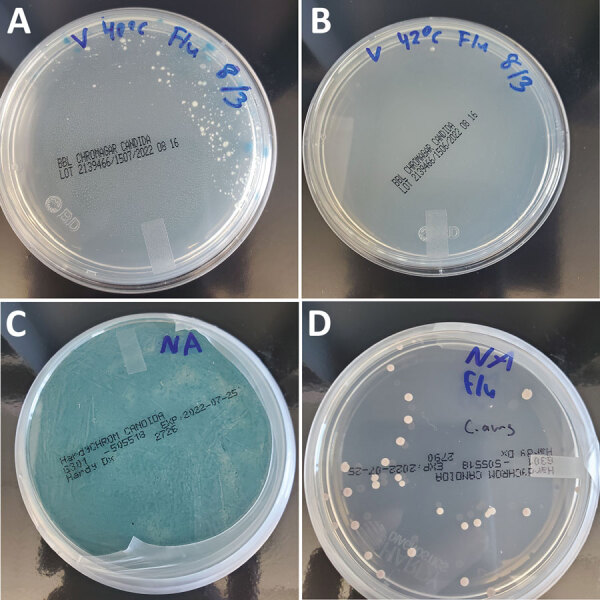
Culture-based isolation of *Candida auris* from wastewater, southern Nevada, USA, 2022, showing the effects of incubation temperature and fluconazole selection on competing organisms. A, B) Incubating the enrichment broth at 42°C (B) instead of 40°C (A) mitigated growth of competing filamentous fungi present in wastewater. C, D) Adding fluconazole to the salt Sabouraud dulcitol broth (SSDB) enrichment broth was essential for recovering *C. auris* from the southern Nevada wastewater samples. We plated 100 μL aliquots of inoculated enrichment broths incubated at 42°C after 2 days on HardyCHROM Candida medium (Hardy Diagnostics, https://hardydiagnostics.com) or BBL CHROMagar Candida medium (Becton, Dickinson, and Co., https://www.bd.com). Inoculation in SSDB without fluconazole resulted in overgrowth of an unidentified competing yeast (C), whereas inoculation in SSDB with fluconazole enabled isolation of *C. auris* colonies (D).

Culture-based recovery of *C. auris* from marine environmental samples has been recently reported (*11*), but isolation from wastewater has not been documented. Following the protocol developed to detect *C. auris* in human specimens, use of SSDB as an enrichment medium was first explored (*9*). However, the selection criteria used for clinical samples in that study proved not stringent enough because fast-growing filamentous fungi and halo- and stress-tolerant *Candida* spp. could easily overgrow during the enrichment step (*12*). Inhibition of competing filamentous fungi, such as *Mucor* spp. and *Geotrichum* spp. (*6*), was mitigated by increasing the incubation temperature of the enrichment broth from 40°C (suggested temperature for patient colonization screening) to 42°C (Figure 1, panels A, B). Suppressing halo- and stress-tolerant *Candida* spp. required adding other selective agents to SSDB. Given that modal MIC for fluconazole for *C. auris* isolates is significantly higher than for other *Candida* spp. (*13*), we pursued an azole-based selection strategy. 

**Figure 2:**
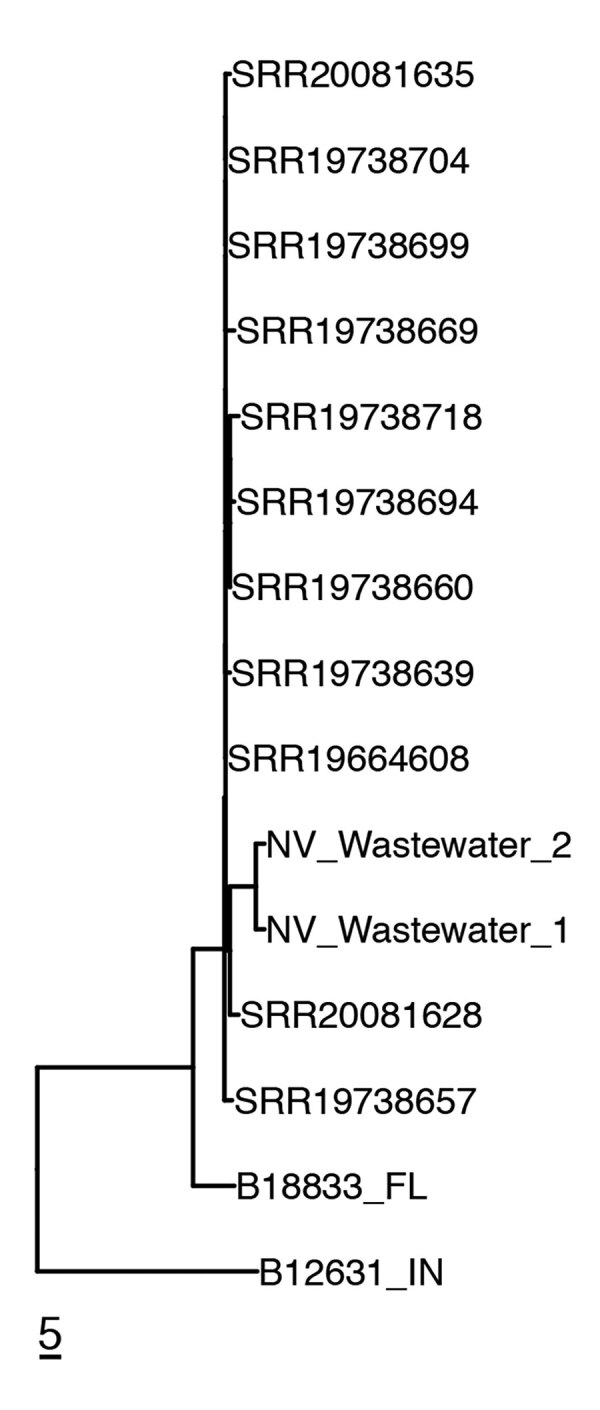
Genetic relatedness of clinical and wastewater isolates identified in the southern Nevada, USA, 2022, to reference clinical isolates. Neighbor-joining phylogenetic tree shows clade III *C. auris* isolates recovered from 3 southern Nevada acute care hospitals (identified by National Center for Biotechnology Information Sequence Read Archive [SRA] accession number) and from the wastewater treatment plants to which they were connected (NV_Wastewater_1 [SRA accession no. SRR21758525] and NV_Wastewater_2[SRA accession no. SRR21758524]). Two unrelated isolates from Florida and Indiana are included in the tree: B188833_FL (SRA accession no. SRR12526241) and B12631_IN (SRA accession no. SRR7909359). Scale bar indicates single-nucleotide polymorphisms.

Preliminary experiments using samples from wastewater spiked with a fluconazole-resistant clinical strain of *C. auris* confirmed that adding fluconazole at a final concentration of 8 μg/mL suppressed growth of nontarget *Candida* spp. while ensuring recovery of *C. auris* (data not shown). We then prospectively validated this approach using wastewater samples collected in southern Nevada, which was experiencing an intense *C. auris* outbreak. Of the 8 samples we collected and analyzed during May 23–June 13, we recovered well-isolated *C. auris* colonies from a single WWTP1 sample from May 23 using SSDB broth enriched in the presence of fluconazole (Figure 1, panel D). Without azole selection, the target organism was overgrown by an unidentified yeast species (Figure 1, panel C). Of note, genomic analysis of 2 separate wastewater-derived *C. auris* isolates revealed phylogenetic relatedness to clade III isolates identified in 3 acute care hospitals within the WWTP1 sewershed (Figure 2). The 2 wastewater isolate genomes differed by 4 SNPs from each other and by 13–20 SNPs from the outbreak-associated isolates from the acute care hospitals (Figure 2). 

## Conclusions 

We report recovery of *C. auris* isolates from wastewater that demonstrated an epidemiologic link to healthcare facilities within the WWTP sewershed in southern Nevada. Although our findings highlight the potential utility of community-level wastewater surveillance for *C. auris*, the methods and the data presented here represent only early-stage implementation. More recent prospective testing of samples in Nevada using both culture and quantitative PCR monitoring (D. Gerrity, unpub. data) has shown considerable variability between samples for the presence of competing organisms, which required increased selection stringency. Indeed, in August 2022, we could repeat isolation of *C. auris* from Nevada samples only by increasing the fluconazole concentration to 32 μg/mL (data not shown). This experience illustrates one limitation of the culture-based approach we used in this study: *C. auris* strains with a fluconazole MIC lower than the concentration of the drug in the broth might not be detected. In addition, the actual sensitivity of this surveillance approach is still unknown, because it can be influenced by the degree of competition between organisms during the enrichment step. PCR-based quantification of *C. auris* genome equivalents in Nevada wastewater indicates that culture-based isolation has occurred at organism concentrations as low as ≈100 CFU/mL (D. Gerrity, unpub. data). In addition, the full extent of *C. auris* colonization, transmission within sewersheds, and organism shedding have not been sufficiently studied. Therefore, it remains to be determined whether *C. auris* could be detected in geographic areas by wastewater surveillance before being recognized in clinical settings. Regardless, wastewater surveillance of pooled samples at the community level might effectively complement clinical surveillance of individual patients for detecting and characterizing *C. auris* outbreaks. 
